# The Development of Cytogenetic Maps for Malaria Mosquitoes

**DOI:** 10.3390/insects9030121

**Published:** 2018-09-17

**Authors:** Gleb N. Artemov, Vladimir N. Stegniy, Maria V. Sharakhova, Igor V. Sharakhov

**Affiliations:** 1 Laboratory of Ecology, Genetics and Environmental Protection, Tomsk State University, 36 Lenin Avenue, 634050 Tomsk, Russia; g-artemov@mail.ru (G.N.A.); stegniy@res.tsu.ru (V.N.S.); msharakh@vt.edu (M.V.S.); 2 Department of Entomology, Fralin Life Science Institute, Institute and State University, Blacksburg, VA 24060, USA

**Keywords:** cytogenetic map, chromosome map, malaria mosquitoes, *Anopheles*, polytene chromosomes, *Anopheles beklemishevi*, chromosome map development, photomap, high-pressure technique, chromosome straightening

## Abstract

Anopheline mosquitoes are important vectors of human malaria. Next-generation sequencing opens new opportunities for studies of mosquito genomes to uncover the genetic basis of a *Plasmodium* transmission. Physical mapping of genome sequences to polytene chromosomes significantly improves reference assemblies. High-resolution cytogenetic maps are essential for anchoring genome sequences to chromosomes as well as for studying breakpoints of chromosome rearrangements and chromatin protein localization. Here we describe a detailed pipeline for the development of high-resolution cytogenetic maps using polytene chromosomes of malaria mosquitoes. We apply this workflow to the refinement of the cytogenetic map developed for *Anopheles beklemishevi.*

## 1. Introduction

Mosquito species within the Anopheles genus are the only vectors of human malaria, which caused approximately half a million deaths in 2017 [1]. There are 444 species of *Anopheles* that differ by the ability to transmit *Plasmodium* [2]. The genetic basis of such differences in vector status is still under investigation [3,4,5]. Whole-genome sequencing and the comparative study of the genomes are the most powerful approaches to better understanding of vector competence and its evolution [3,6]. However, genome sequencing alone cannot achieve chromosome-level assemblies, which are important for further genome analysis. Physical mapping of the genome can anchor scaffolds to polytene chromosomes using fluorescence in situ hybridization (FISH) and can map up to 97% of a genome sequence to chromosomes [6,7].

Genome assemblies for 16 species of malaria vector mosquitoes have so far been obtained [6]. Physical mapping in five *Anopheles* species corrected mistakes in genome assemblies [7] as well as discovered a high rate of X chromosome evolution [6] and inter-arm rearrangements in pericentric regions [8]. High-resolution сytogenetic maps are required for physical genome mapping and studies of mosquito chromosome organization and evolution.

The chromosome complement of *Anopheles* mosquitoes consists of three pairs of chromosomes: two sex chromosomes (XX in females and XY in males) and two pairs of submetacentric autosomes. Polytene chromosomes in *Anopheles* are developed in several cell types: salivary glands, Malpighian tubules, midgut and fat body of larvae, and Malpighian tubules and nurse cells of adults. Differences exist in the polytene chromosome quality among tissues and species, which depends on the degree of polyteny and chromatids conjugation. Polytene chromosomes in most tissues of *Anopheles* mosquitoes are joined together in the chromocenter, except for ovarian nurse cell chromosomes of species from the Maculipennis group, where pericentromeric regions separate from each other [9].

The first cytogenetic maps of *Anopheles* polytene chromosomes were developed in the 1960s, and today there are about 100 cytogenetic maps available for different species [10]. The first cytogenetic maps were drawn, making them prone to a subjective interpretation of banding patterns. With the advance of micro-photography techniques, chromosome maps were built from chromosome photo images first on photocards and then using digital photos. Subjectivity is reduced by micro-photo techniques, but conventional photo-maps are usually based on only one chromosome spread. The best cytogenetic map can be developed from straightened chromosome images created by combining the most typical high-resolution regions into a single collage. This approach started with paper photo collages when chromosome images were cut from paper photos, arranged and glued on a paper sheet, and then photographed again [11,12,13]. Many details can be lost in a double-photography process. Chromosome staining can also reduce details in the banding pattern. This disadvantage has been overcome by phase-contrast microscopy, which is the most appropriate approach for detailed visualization of the polytene chromosome banding pattern [14,15,16,17].

A modern chromosome map development approach uses digital phase-contrast chromosome images to straighten and combine typical chromosome regions utilizing computer software. Using this approach, high-resolution chromosome maps for ten *Anopheles* species have been developed, including *An. albimanus* [7,17], *An. funestus* [14,18], *An. stephensi* [16], *An. gambiae* [15], *An. darling* [19,20], *An. nili* [21], *An. sinensis* [22], *An. atroparvus* [8,23], *An. lesteri* [24], *An. beklemishevi* [25], and *An. sacharovi* [26]. Some of these chromosome maps were used for physical mapping, thus, improving genome references for *An. gambiae* [15], *An. stephensi* [27], *An. albimanus* [7], *An. atroparvus* [8,23], and *An. sinensis* [28]. Here we describe in detail a pipeline for chromosome photomap development based on modern approaches. We apply it to the refinement of the cytogenetic map for *An. beklemishevi* published earlier [25].

## 2. Materials and Methods

### 2.1. Overview of the High-Resolution Cytogenetic Map Development

The goal of high-resolution cytogenetic maps is to make chromosomes and chromosome regions easily identifiable. Toward this goal, we propose three major steps: (1) preparation of chromosome arms for straightening; (2) chromosome arms straightening, trimming, and processing (brightness and contrast adjustment, chromosome regions combination and substitution, etc.); and (3) dividing chromosomes by numbered and lettered regions.

Processing the chromosome map development starts with squashed (or high-pressure squashed) chromosome preparations and microscopy of no less than 50 chromosome squashes. Only high-quality images of the chromosomes with typical banding patterns, chromosome edge profiles, and centromere and telomere ends are included in a chromosome map. These features are defined as typical if they are present in more than half of chromosome preparations. Chromosome straightening aimed to organize all chromosome bands in a parallel orientation to each other (a barcode-like pattern) and to align the chromosomes along the central axis. Chromosomes must preserve the native and typical (reproducible) morphology of a banding pattern, which is improved by straightening. Trimming of chromosome images reveals the shape of the chromosome, which is important for identifying some chromosome landmarks. No less than three best-straightened chromosomes must be combined in the final cytogenetic map.

A chromosome map reflects the proportions of chromosome arms and position of landmarks relative to the chromosome ends. The average measurements of chromosome arm length and the distance from the telomere end to a landmark are calculated for the relative chromosome length adjustment and placement of a landmark to the proper position.

The final step of the cytogenetic map development is to divide chromosomes by both numbered divisions and lettered subdivisions according to existing chromosome maps or by relying on a basic principle of this procedure described below. The boundaries between divisions and subdivisions are always placed to the borders between bands and interbands.

### 2.2. Tissue Fixation and Chromosome Preparation

For polytene chromosome preparation, salivary gland cells of larvae or ovarian nurse cells of adults of *An. beklemishevi*, *An. sacharovi* and *An. quadrimaculatus* were used. For salivary glands fixation, a whole 4th instar larvae were directly put in Carnoy’s solution (3 volumes of 100% ethanol and 1 volume of glacial acetic acid). For ovarian nurse cells fixation, half-gravid females were dissected and their ovaries were transferred into Carnoy’s solution. Material was stored in Carnoy’s at −20 °C.

Salivary glands or 15–20 ovarian follicles were dissected and put onto a slide containing a drop of 50% propionic acid for 5 min. A filter paper was used to remove the liquid, and a new drop of 50% propionic acid was added. The material was covered by a coverslip, then by a piece of filter paper, and squashed by tapping it with a pencil eraser tip. High-pressure squashed chromosome preparations were prepared following protocols adapted for *An. gambiae* [15,29].

### 2.3. Microscopy, Chromosome Measurement, and Image Processing

Chromosomes were viewed using AxioImager A1 (Carl Zeiss, OPTEC, Novosibirsk, Russia) under phase-contrast with a 100× objective. High-resolution chromosome images were captured by a CCD camera MrC5 (Carl Zeiss, OPTEC, Novosibirsk, Russia) and AxioVision 4.8.2 software (Carl Zeiss, OPTEC, Novosibirsk, Russia) using a 2584 × 1936 pixels multicolor camera mode. Chromosomes were measured using the “Measurement” option in the AxioVision 4.8.2 software package. Chromosome images were further manipulated and modified using Photoshop CS6 (Adobe Systems Incorporated, San Jose, CA, US).

### 2.4. Image Acquisition

**Figure 1:**
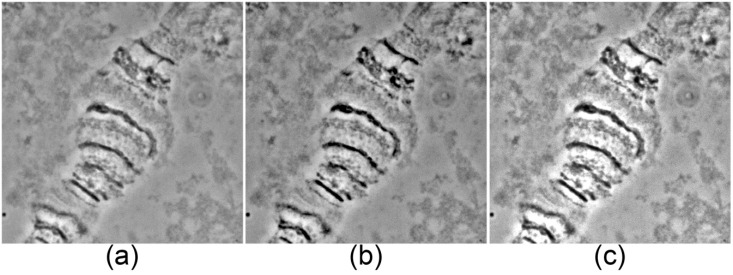
Levels of shadows and highlights in images of a 3R arm chromosome region of *An. quadrimaculatus* from ovarian nurse cells. (**a**) Appropriate levels of shadows and highlights; (**b**) over-shadowed levels; (**c**) over-highlighted levels.

Chromosome images can be obtained with any available software compatible with a microscope digital camera. High-resolution images should be created using a 100× objective and a camera mode of no less than 2584 × 1936 pixels. Shading, white balance, and exposure must be adjusted prior to photographing (Figure 1). It is important to avoid over-contrasted (Figure 1b) and over-bleached images (Figure 1c), which will be impossible to correct during further steps.

**Figure 2:**
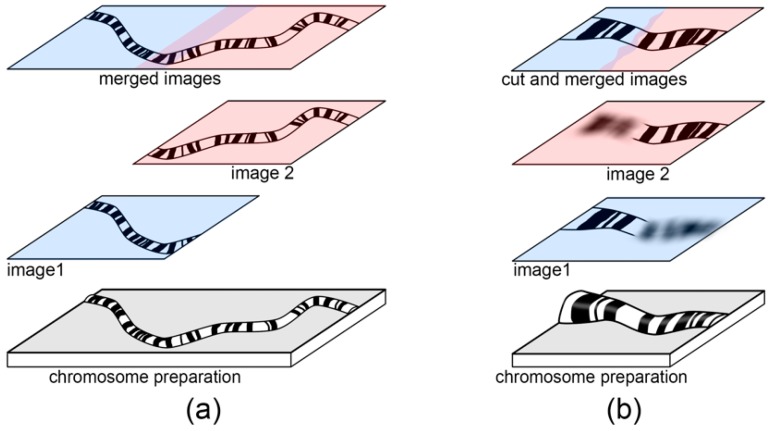
Images merging scheme. (**a**) A chromosome region from a single microscope field; (**b**) a chromosome region in different focal planes. Images 1 and 2 are taken from two different and overlapped microscope fields (**a**) or from two different focal planes (**b**). Merged images performed by a “Merge Layer” function (**a**) or by cutting unfocused fragments of images with a “Lasso Tool” prior to merging layers (**b**). All manipulation tool references relate to Photoshop CS6.

Usually, polytene chromosomes are too long to fit into the view of the objective lens field at 100× magnification. This can be a challenge for chromosome arm identification, chromosome map measurement, and chromosome straightening. To overcome this difficulty, a set of overlapping images of different chromosome regions should be obtained (Figure 2a). In case the same chromosome spreads are not completely flat in a preparation, several images of the same chromosome spread with different focus planes must be obtained (Figure 2b).

Only images of chromosomes (or regions) with a good-quality banding pattern should be chosen for the map development. Good-quality chromosomes (or regions) have the following 5 characteristics: (1) chromosomes are well separated from each other and do not overlap; (2) chromosomes are not stretched; (3) borders between bands and interbands are well-defined; (4) thin bands are visible; and (5) chromosomes and chromosome landmarks have typical reproducible structures and should be well-polytenized.

### 2.5. Image Selection and Processing

For chromosome map development, we usually have a set of images—some with a single chromosome, others with 2 or several different chromosomes or even parts of chromosomes. If a chromosome is on 2 or more images, these images must be merged prior to further processing. For this purpose, copies of such images must be moved as new layers to a separate sheet (Figure 2). For a precise chromosome assembly without gaps, the images of the chromosome must overlap. Before merging the layers, the edges of the front layers must be smoothed with a smooth brush such as a “Smooth Tool” or an “Erase Tool” to keep the edge of the overlapping layer visible.

If a whole chromosome is not in the same focus plane on a preparation, several images of different chromosome parts must be obtained. At this step, all images are moved to a new sheet and are overlapped as in the previous case. Then, each in-focus image of the chromosome regions is preserved, while out-of-focus parts are removed. Undesirable image areas should be selected with the “Lasso Tool” or “Rectangular Marquee Tool” and deleted. The edges of images can be processed with “Smooth Tool” or by “Eraser Tool” to achieve a gradual transition between the layers.

After processing, the appropriate chromosome images must be cleaned of excess background areas and desaturated if they were colored. Chromosomes must be contoured by a “Lasso Tool” and indented from the chromosome edge by approximately half of the chromosome width so that each chromosome is surrounded by a stripe of the background. This step is crucial for chromosome straightening, as adjacent parts of chromosome edges cannot coincide and must be trimmed (see below). A chromosome region selected by the “Lasso Tool” should be transposed to a new layer and moved to a new sheet where all chromosome arm images are stored. Some layers with chromosome images will be used as templates for straightening chromosomes in the following steps.

### 2.6. Chromosome Landmarks Layout and Work Area Preparation

**Figure 3:**
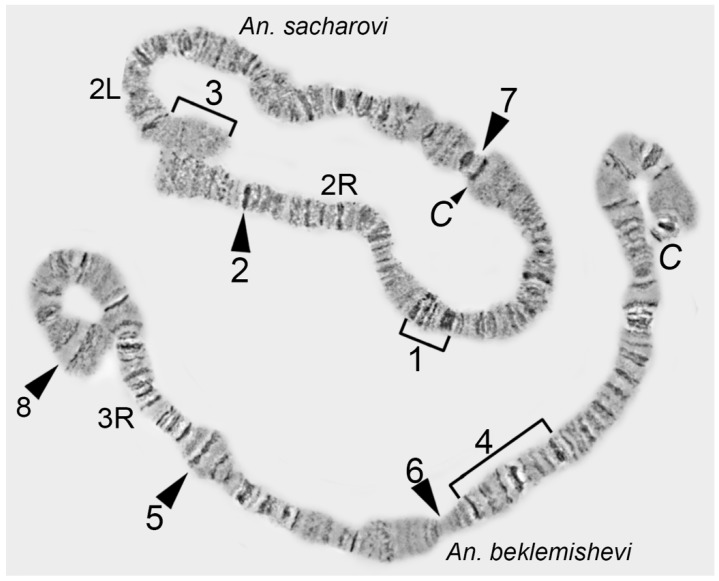
Examples of landmark regions in chromosome 2 of *An. sacharovi* and 3R arm of *An. Beklemishevi*: 1—a set of irregular bands; 2—a dark single band; 3—a region with long interbands; 4—a region with a set of 3 dark bands; 5—a protrusion; 6—an indentation; 7—an indentation surrounded by two dark bands; and, 8—a large interband restricted by 2 bands.

Chromosome arms and regions are defined by regions with unusual structures—i.e., chromosome landmarks. Landmarks are characterized by the following features: (1) irregular banding pattern (a batch of juxtaposed bands or large interband); (2) unusually wide bands; (3) bands of high density (darker than other bands); and, (4) protrusions and hollows on the chromosome edge profile (Figure 3).

**Figure 4:**
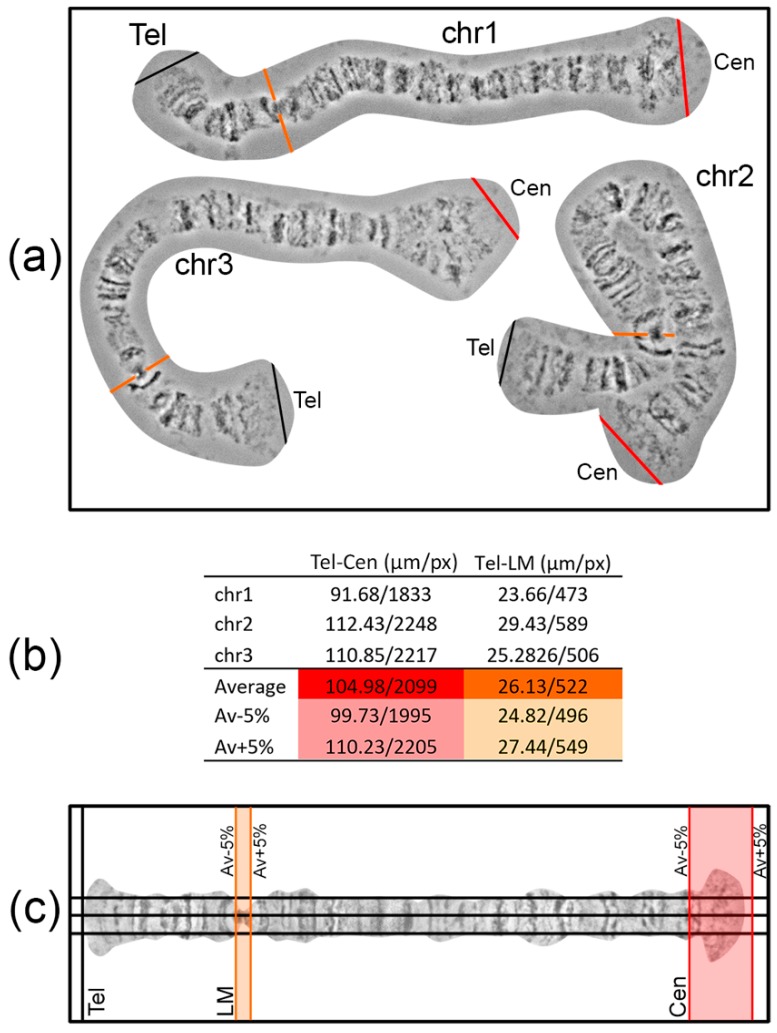
A workflow of the work area layout of the 3L arm of *An. sacharovi* nurse cells polytene chromosomes. (**a**) Templates of 3L arms, named chr1, chr2, chr3, Tel—telomere (black line), Cen—centromere (red line), with the “bird eye” landmark arrowed by orange line; (**b**) table of the distances between telomere and centromere ends (Tel-Cen), and between telomere end and the “bird eye” landmark (Tel-LM) of 3 arms examples and their average value, with 5% error margins, in microns and pixels; (**c**) work area layout: telomere end (Tel) marked by vertical black guideline, centromere end (Cen) and “bird eye” landmark (LM) positions marked as colored areas limited by the pairs of vertical lines, where the centromere end and the landmark must appear.

Landmarks are important for identifying the chromosome arm and regions surrounding landmarks. For chromosome map development, it is crucial to select landmark regions and to define their position relative to chromosome arm ends. Most often, peritelomeric and pericentromeric regions are sufficient landmarks for identification of chromosome arms. In many cases, one or several landmarks within a chromosome arm can be selected. For instance, this can be useful for determining the orientation of inversions. The position of such “internal” landmark regions is calculated relative to the telomeric end in order to establish its proper location on a chromosome map. It is advisable to make measurements of the distance between the marginal telomere point to a certain point on the landmark region using no less than 3 different high-quality unstretched chromosome images. The average value of the measurements and fitted 5% error will create a frame in which landmarks have to appear on a chromosome map (Figure 4).

To layout a work area where a chromosome map will be developed, 3 horizontal and several vertical guidelines are created (Main menu > View > New guide in Photoshop). Three horizontal guidelines should be at an equal distance from each other. The central guideline is the axis of a future straightened chromosome; the other 2 are auxiliary guidelines, situated at middle distance from the chromosome axis to the chromosome edge. These 3 horizontal guidelines facilitate the establishment of band and interband positions relative to the chromosome axis during chromosome straightening. The first vertical guide is set on the left side of the sheet where the telomere end will appear. On the right side of the sheet, a pair of vertical guidelines is placed to mark the borders of a centromere end. The positions of these guidelines are calculated as average chromosome arm length ± 5% error (Figure 4). The aim of the layout is to predict the locations of landmarks and a centromere end on a future chromosome map.

### 2.7. Chromosome Straightening

**Figure 5:**
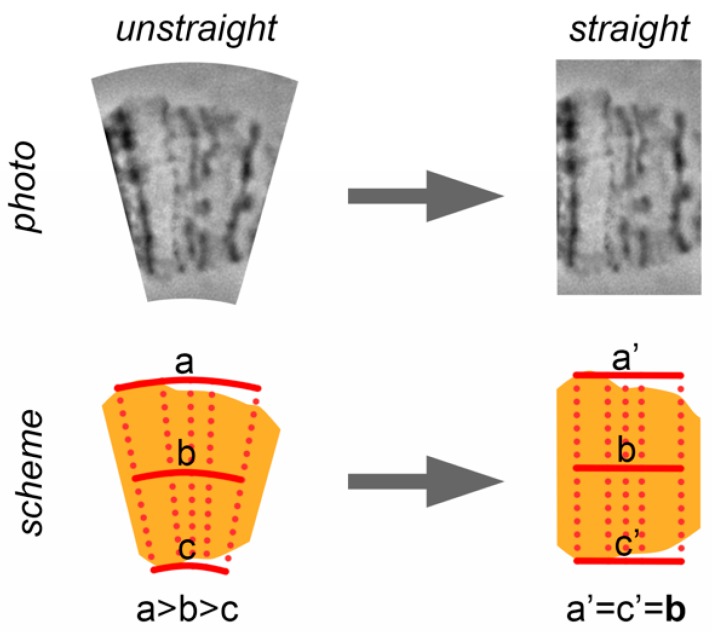
A principle of chromosome straightening. The distances between marginal points of the different bands (a, c) became equal to the distance between the axial points of the bands (b) after chromosome straightening.

Even the best chromosome preparations have minor curves that should be straightened to produce chromosome maps. Usually curves do not deform the band shape. Instead, curves transform the interband shape. When a chromosome is aligned, the distance between the adjacent bands is approximately the same as the full width of a chromosome. When a chromosome is curved, the distance between bands on the major radius of the curve becomes bigger and, on the minor radius of the curve, becomes smaller, than those in a straight chromosome. The natural distance between bands is maintained only on the axial part of the chromosome (Figure 5).

Thus, chromosome straightening is a procedure of adjusting the distance between the marginal parts of chromosome bands to the distance between axial parts of the chromosome bands (Figure 5).

**Figure 6:**
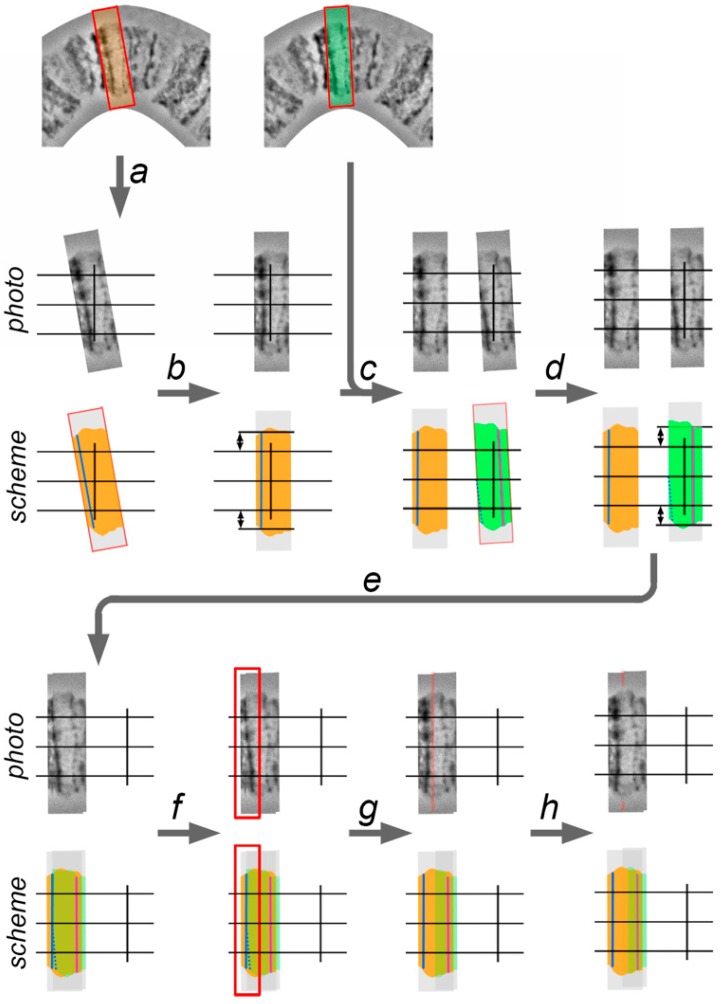
A workflow of chromosome straightening; example for the region with two bands: a—selection, which includes two adjacent bands, using “Lasso Tool” or “Rectangular Marquee Tool,” copying and moving the new layer with “Move Tool” to the work field; b—rotation and movement of the new layer (orange area) with “Move tool”, applying the “Transform” function, and adjustment of the first band (blue line), vertically, relative to the vertical guideline; the distance between marginal points of the band is the same relative to the outer horizontal lines; c—new selections on the template chromosome image, which include the whole second band and the whole, or at least part, of the first band (dotted blue line); copying selection and moving the new layer to the work field; d—rotation and movement of the second layer (green area) with “Move tool”, applying “Transform” function, and adjustment of the second band (magenta line), vertically, relative to the vertical guideline; the distance between marginal points of the band is the same relative to the outer horizontal lines; e—the second layer is brought to the front and overlapped with the first layer using “Move Tool”; the axial part of the first band on the second layer coincides with the axial part of the first band of the first layer (varying the opacity of the second layer is useful); f—a new selection in the work field, which includes the first band of the second layer (blue dotted line) and a region of the second layer which covers the first band of the first layer (blue line) using “Lasso Tool” or “Rectangular Marquee Tool”; g—deletion of selection; h—blurring the left border of the second layer, using “Eraser Tool” with smooth border brush or “Blur Tool”, to make a gradual transition from the first layer to the second layer.

All bands on the chromosome map have to be set perpendicular to the chromosome axis and set to the native distance between them. Moreover, the central point of each band has to coincide with the chromosome’s axis. To control parallelism between all bands and its position on the chromosome axis, vertical guidelines are created (Figure 6).

Chromosome straightening is performed from left to right (from the telomere to the centromere) and consists of 4 steps: (1) cutting the layer with a band (with surrounding interbands and, sometimes, other bands) from the template chromosome image; (2) adjusting the band to a vertical orientation and establishing it relative to the chromosome axis; (3) establishing a band on the proper distance relative to the previous band; and, (4) retouching the edges of 2 adjacent layers (Figure 6). The techniques of this process are described below.

1. Using the “Lasso Tool” or “Rectangular Marquee Tool”, the selection, which includes current (second) and previous (first) adjacent bands, is made on the template chromosome image. The copy of the new layer is created from the selection and transposed to the work area by “Move Tool.”

2. The second (current) band is aligned according to the vertical guideline to make it perpendicular to the chromosome axis. This is performed by rotating the layer with the use the transformation function.

3. The layer with the second band is moved to the front and is overlapped with the first layer by using the “Move Tool”. The axial part of the first band copy on the second layer must coincide with the axial part of the first band. This placement is required in order to follow the native distance between two adjacent bands. After that, some parts of the first layers can be covered by the second layer, as this part of the second layer must be deleted. A new selection in the work area, which includes the first band on the second layer and a region of the second layer which covers the first band on the first layer, is made using the “Lasso Tool” or “Rectangular Marquee Tool”, and then deleted.

4. The left edge of the second layer is sharp and makes the transition between the first layer to the second layer visible. To conceal the transition, the left edge of the second layer must be processed using an “Eraser Tool” with a smooth border brush or by the “Blur Tool.”

Iteration of these four steps applied to each band from left to right in a curved chromosome region makes it completely straight.

### 2.8. Chromosome Regions Combination and Substitution

A perfect whole-arm chromosome image is almost impossible to obtain. After the straightening procedure, some regions of chromosomes have deficiencies that must be edited. We created at least 3 native view variants of straightening chromosome arms because different regions could have a different quality in each variant. Such regions are selected and combined to make the best result. Selected layers are copied using “Move Tool” in a new work area, where the finished variant of a chromosome arm will be developed. Centering, aligning, and retouching procedures are the same as described above.

Centromere and telomere ends are important landmarks for a chromosome arm discrimination, and they should be thoroughly developed on the chromosome map. Because of their heterochromatic nature, these regions usually have no typical banding pattern. Similar to other regions, centromere and telomere ends can have the most typical variants. The telomere and the centromere ends are usually substituted from an appropriate template image that does not need straightening. In the chromosomes of nurse cells, homologues are often asynaptic at the centromere end. These homologues typically have different degrees of stretching and varying banding patterns and curves. Therefore, the centromere end cannot be transposed as a whole to a chromosome map. To resolve this problem, one homologue from a pair is processed and transposed to the chromosome map. Then, it is copied to a separate layer and flipped. In this case, the angle between homologues and the length of the asynaptic centromere region must have the most typical characteristics for this region.

### 2.9. Chromosome Trimming

One of the most crucial steps of chromosome map development is the trimming of the background from the straightened chromosome arm. The chromosome edge profile, along with the banding pattern, is an important characteristic of chromosome and region identification, and often serves as a landmark. A straightened chromosome arm is surrounded by a background from the template chromosome images. These parts of the layers are deleted to uncover a chromosome edge profile. Usually, marginal points of the bands are at the bottom of the indentations, and the interbands (or puffs and diffuse heterochromatin) form protrusions. However, there are cases which depart from this rule, and they can also be important landmarks.

**Figure 7:**
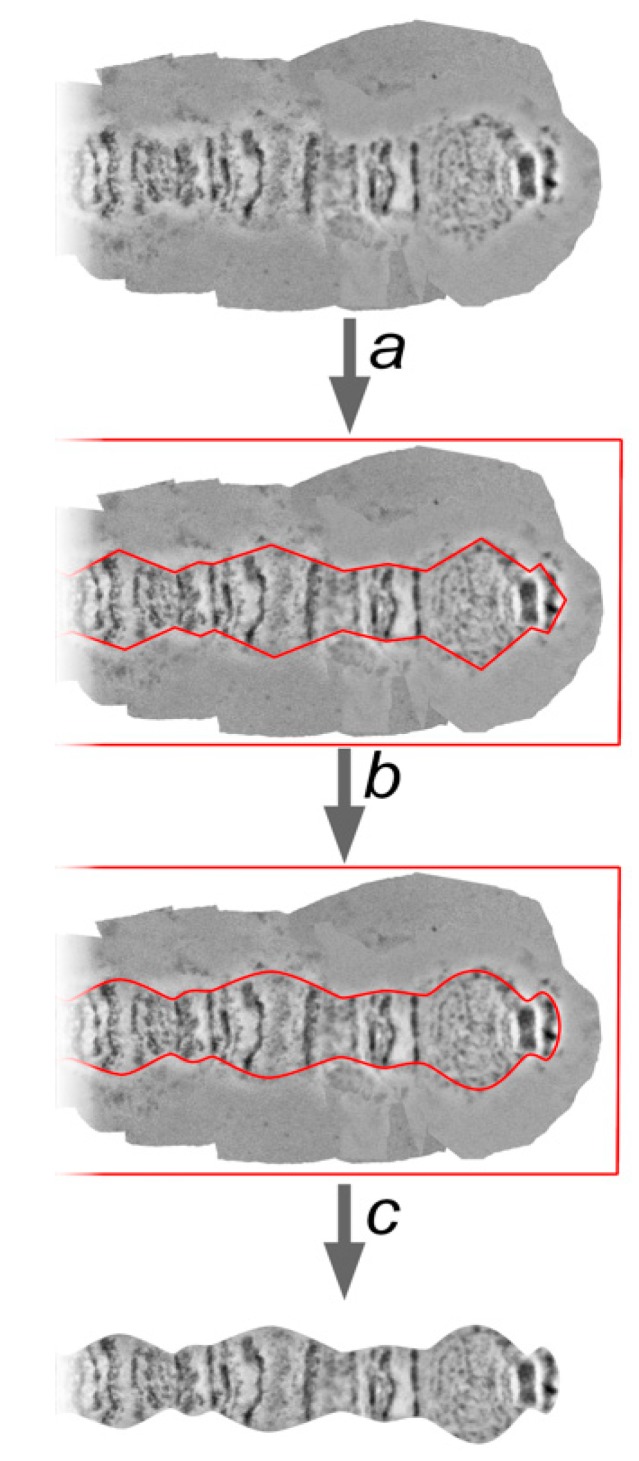
Trimming of a chromosome arm map (the pericentromeric region of 3R arm of *An. beklemishevi* nurse cells as an example): a—making the path, using “Path Tool”, and marking protuberances and hollows of chromosome edge profile (red line); b—smoothing angles using “Convert Point Tool” (red line); and, c—making the selection from the path and deleting the background area.

The procedure of trimming must not change a landmark’s features. For this reason, landmarks, as well as the thinnest and the widest regions on the chromosome arm, must receive special attention. Following these observations, the sequence of protrusions and indentations on the chromosome edge profile is marked by a “Pen Tool”. These marginal points are then curved by a “Convert Point Tool” in accordance with the native shape of each chromosome region (Figure 7).

The “Path” needs to be transformed into the “Selection” using the “Make selection” function, then the selection needs to be inverted with the “Select inverse” function and the background must be deleted leaving layers containing the straightened chromosome.

### 2.10. Final Corrections

Final corrections are performed on a separate sheet where all of the stretched chromosome arms are collected.

#### 2.10.1. Brightness and Contrast Adjustment

Brightness and contrast can be changed at the step of image processing, but the excess background can impede appropriate image edition. After trimming, only chromosome images will be processed. Brightness and contrast adjustments are important, not only for general image processing, but also for modifying landmark regions according to their native view.

Usually desirable brightness and contrast values are achieved by adjusting tones in the levels menu (Image > Adjustments > Levels). Dragging the black and white points to the left and right edges of the histogram palette darkens the shadows and brightens the highlights. Mid-tone points can be adjusted also, but over-shadowing and over-highlighting must be avoided (Figure 1).

#### 2.10.2. Relative Chromosome Length Position Adjustment

Relative chromosome length and centromere indexes are very important karyotype characteristics, and a chromosome map must reflect them. To avoid differences in the levels of polyteny and chromatin condensation, 20–30 images of the chromosome sets must be obtained. These images are used for calculating relative chromosome lengths and centromere indexes. For each chromosome set, the lengths of arms are measured, and relative chromosome lengths and centromere indexes are calculated. The average values of these characteristics are used to adjust straightened chromosome arms.

After being assembled, centromere ends have to be found within the frame, which means that the average chromosome length must be within a ±5% error. However, the current lengths of individual chromosome maps do not completely correspond to the calculated proportions. Using the shortest chromosome arm as a standard, new values for each chromosome arm map length are calculated. The lengths of each chromosome arm layer are adjusted to the new length values using “Move Tool”. Minor corrections to the width of the chromosomes are also occasionally required.

#### 2.10.3. Chromosome Map Structure

Chromosome arm maps are arranged on a canvas according to these rules:

1. The telomere ends must be right oriented, and the centromere ends must be left oriented.
2. Sex chromosomes are located on the top of the map. The remaining chromosome arms are positioned according to the division number, starting from the right arm.
3. All arms must be left-edge-justified. The distances between chromosome arms should be sufficient to allow the numbering and lettering of divisions and subdivisions.

### 2.11. Chromosome Regions Division and Nomenclature

To reference certain regions, chromosome arms are divided into numbered divisions and lettered subdivisions. Divisions (subdivisions) should correspond to painted or photo maps of the same cell-type chromosomes for succession. It may be difficult in chromosome map subdivisions to do this for different cell types because of inter-tissue differences in banding patterns. However, it should be done for divisions, at least.

**Figure 8:**
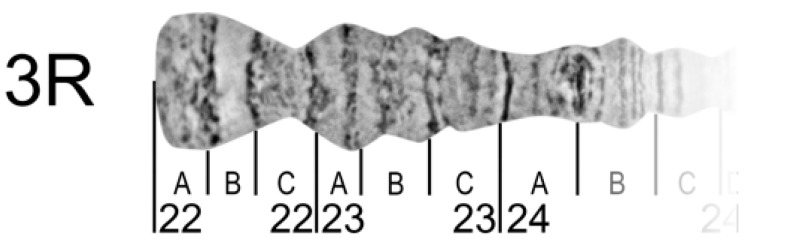
Chromosome division, numbering, and lettering (peritelomeric region of 3R *An. beklemishevi* as an example).

The borders of divisions (subdivisions) must always mark the borders of bands and interbands (Figure 8).

The result of a boundary setting is to define the band or interband to a certain division (subdivision). Each division (subdivision) contains at least one band. The borders of divisions and subdivisions are indicated by vertical lines. Each border is drawn from the chromosome edge exactly opposite the band-interband border below the straightened chromosome image. Division lines are longer than subdivision lines (Figure 8).

Chromosome names are aligned with the axis of each chromosome arm and are placed near the telomere end. In the final step, the numbering and lettering of chromosome regions should follow the nomenclature of existing chromosome maps for certain species. In the right arms, numeration and lettering are from the telomere to the centromere end of the chromosome arm (from left to right) but, in the left arms, from the centromere to the telomere ends (from right to left). This approach provides continuity in numbering and lettering of regions for the whole chromosome.

## 3. Results

**Figure 9:**
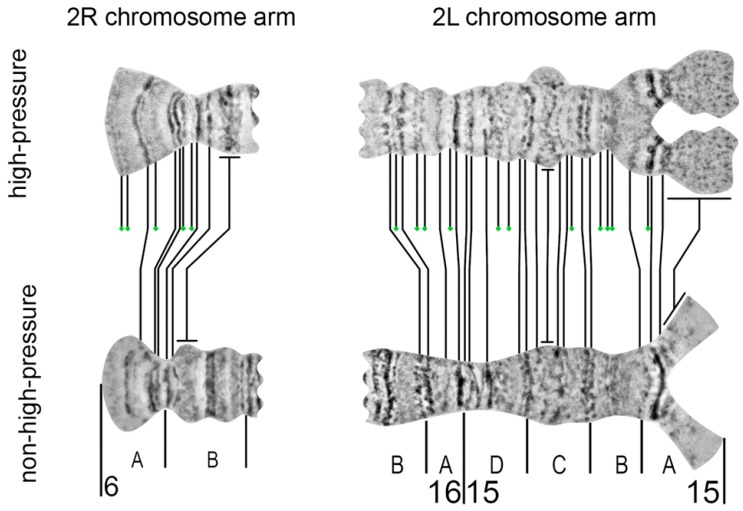
Comparison of the high-pressure (**top**) and non-high-pressure (**bottom**) maps for 6A–6B region in the 2R arm and 15A–16A in the 2L arm of nurse cell chromosomes of *An. beklemishevi*. The new bands in the high-pressure map are labeled by green circles.

Following the procedures described above and high-pressure techniques [15,29,30,31], we upgraded the cytogenetic maps of two regions of nurse cell chromosomes of *An. beklemishevi* [29]: regions 6A–6B in the 2R arm (a telomeric end) and regions 15A–16A in the 2L arm (a pericentromeric end). The main result of the high-pressure application was the discovery of several minor bands, which are absent on the non-high-pressure chromosome map: 5 bands in region 6A–6B and 11 bands in region 15A–16A (Figure 9).

For example, two fine bands at the end of the peritelomeric region in the 2R arm became visible only after high-pressure squashing. Another important result was the visualization of near-regular banding-like structure of the pericentric diffuse chromatin in region 15A of the 2L arm. After high-pressure squashing, the chromatin fibers and numerous aggregates corresponding to heterochromatic “bands” are revealed.

## 4. Discussion

Here we describe the basic principles and detailed techniques of a high-resolution cytogenetic map’s development for malaria mosquitoes’ polytene chromosomes. We used high-resolution digital images of the squashed preparation of unstained polytene chromosomes to develop the map. Such images preserve fine details of the chromosome banding pattern. The major principle of map development is to reproduce the native structure of the polytene chromosomes and to make chromosome arms and regions on the map easily identifiable. In our map-developing approach, this principle is achieved by straightening chromosomes and by paying special attention to the position and morphology of landmarks. The straightening procedure is performed by aligning the chromosome bands sequentially, making them parallel and barcode-like. Landmarks and their positions facilitate identification of the chromosome arms and surrounding internal regions. Chromosome shape is a very important landmark, and our approach reflects the chromosome edge profile. Thus, in defining chromosome regions, an investigator relies not only on the banding pattern but also on the sequence of protrusions and on the indentations of the chromosomes.

Chromosomal divisions and nomenclature, in general, are adopted from the tradition for dipteran polytene chromosomes. The divisions are indicated by numbers, and subdivision names are spelt with capital letters [31,32,33]. However, we have changed the sequence of subdivision letters in the left autosomal arms to follow the sequence of numbers from centromeres to telomeres.

The chromosomal position of DNA markers after FISH can be identified using cytogenetic maps. Therefore, genome physical mapping can anchor sequencing scaffolds to polytene chromosomes during the mapping procedure. Chromosome maps developed using this strategy have been successfully used for physical mapping of the five anopheline genomes: *An. gambiae* [15], *An. stephensi* [27], *An. albimanus* [7], *An. atroparvus* [8,23], and *An. sinensis* [28].

The quality of polytene chromosomes and cytogenetic maps determines the resolution of the physical mapping. Resolution is important for identifying the direction of supercontigs mapped by FISH, when the beginning and the end of each supercontig is labelled by different fluorochromes. If the structure of the polytene chromosome is “friable”, and the borders between bands and interbands are fuzzy, DNA markers of small supercontigs will overlap. Sometimes the resolution of genome physical mapping can be improved by a high-pressure technique [15,29,30] which makes the banding pattern clearer and many new small bands visible.

Here we applied a high-pressure technique to refine two regions on the chromosome map of *An. beklemishevi*. This approach makes visible an additional 45% of bands in two subdivisions of the 2R arm and 38% of bands in five subdivisions of the 2L arm. The high-pressure method reveals a previously invisible band-like structure in heterochromatic regions. The fan structure of fibrils in region 15A has been confirmed by FISH of 15A-specific micro-dissected DNA-probe with nurse cell chromosomes of *An. beklemishevi* [34]. The high-pressure technique is required to make a detailed map of heterochromatin regions of polytene chromosomes, which often have a diffuse structure [35].

Despite its advantages, the high-pressure technique can introduce deformation in the chromosome structure. Chromosome overstretching and band over-squashing are common outputs of this technique. The approach results in appropriate squashing of only some chromosomes on a preparation or some regions within a chromosome arm. Moreover, it can transform morphology of some regions to make it unnatural, as it was shown with a puffed region in 15C; after high-pressure squashing, it made protrusions, which are absent in regular-pressure chromosome preparations (Figure 9). Thus, the high-pressure technique is not suitable for routine physical mapping. The high-pressure chromosome preparations may be used for improvement of specific chromosomal regions when precise mapping is required.

## 5. Conclusions

High-resolution cytogenetic maps are essential for anchoring genome sequences to chromosomes as well as for studying chromosome organization and evolution. We developed a detailed pipeline for the construction of high-resolution cytogenetic maps and applied this workflow to the refinement of the cytogenetic map for *An. beklemishevi*. The method presented here can be used to develop high-resolution cytogenetic maps for other *Anopheles* mosquitoes, as well as for other dipteran insects with polytene chromosomes.
